# Estimation of minimal detectable change in the 10-meter walking test for patients with stroke: a study stratified by gait speed

**DOI:** 10.3389/fneur.2023.1219505

**Published:** 2023-07-19

**Authors:** Yuichiro Hosoi, Takayuki Kamimoto, Katsuya Sakai, Masanari Yamada, Michiyuki Kawakami

**Affiliations:** ^1^Department of Rehabilitation Medicine, Keio University School of Medicine, Tokyo, Japan; ^2^Department of Physical Therapy, Faculty of Health Sciences, Tokyo Metropolitan University, Tokyo, Japan; ^3^Department of Rehabilitation, Ukai Rehabilitation Hospital, Aichi, Japan

**Keywords:** patients with stroke, 10MWT, gait time, gait speed, reliability, MDC

## Abstract

**Objective:**

This study aimed to classify and calculate the minimal detectable changes (MDC) in gait time and gait speed in a 10-meter walking test (10MWT) in patients with stroke classified according to their gait speed.

**Methods:**

The participants were 84 patients with stroke. Their gait times were measured twice each at their comfortable gait speed (CGS) and maximum gait speed (MGS) on a 10-meter straight track, and gait speed was calculated using gait time. Participants were assigned to three speed groups based on their CGS: low-speed (<0.4 m/s; *n* = 19); moderate-speed (0.4–0.8 m/s; *n* = 29); and high-speed (>0.8 m/s; *n* = 36). For each group, first and second retest reliability and MDC of CGS and MGS were calculated using gait time and gait speed in the 10MWT.

**Results:**

MDCs in the 10MWT at CGS were: low-speed group, gait time 5.25 s, gait speed 0.05 m/s; moderate-speed group, gait time 2.83 s, gait speed 0.11 m/s; and high-speed group, gait time 1.58 s, gait speed 0.21 m/s. MDCs in the 10MWT at MGS were: low-speed group, gait time 7.26 s, gait speed 0.04 m/s; moderate-speed group, gait time 2.48 s, gait speed 0.12 m/s; and high-speed group, gait time 1.28 s, gait speed 0.19 m/s.

**Conclusion:**

Since the MDC of gait speed and gait time differ depending on the participant’s gait speed, it is necessary to interpret the results according to the participant’s gait speed when judging the effectiveness of therapeutic interventions.

## 1. Introduction

Patients with stroke frequently have gait impairments, such as low gait speed, low endurance, and low gait independence (1). Following a stroke, approximately 70% of individuals older than 65 years of age regain independent gait within 6 months (2), but only 30% of gait speeds exceed 0.8 m/s following inpatient rehabilitation (3). Previous studies have reported an average gait speed of 0.36 m/s in patients in the subacute stroke phase (4), and 0.56 m/s in patients in the chronic stroke phase (5). Furthermore, the incidence of falls within 1 year of stroke onset has been reported to be 73% (6). Therefore, recovery of gait ability in patients with stroke is an important and major goal of rehabilitation (7, 8).

The 10-meter walking test (10MWT) is one of the most common methods for assessing gait ability (9), that can be easily and quickly evaluated in the laboratory as well as at the clinical site (10), and has been reported as being reliable in many reports (11–14). Furthermore, since the 10MWT has been reported to be associated with motor function (15), health-related quality of life (16), and predictors of survival (17), capturing changes in gait speed is considered very important (18).

In recent years, although many studies have reported the importance of assessing changes in gait speed and intervention effects in patients with stroke in terms of the minimum detectable change (MDC) (5, 19, 20), the reported MDC is highly variable. Patients with stroke have large variability in gait speed due to the influence of motor function and gait ability (21). Furthermore, according to previous studies, there is an association between motor function and gait ability in patients with stroke, with low motor function typically observed with low gait speeds (15, 22). In addition, calculation of gait speed is often performed using the gait time obtained in the 10MWT. Several previous studies using the 10MWT have assessed the observed gait time, rather than gait speed, as the outcome measure (23–26), because it is easier to use gait time measured in the 10MWT at clinical sites. However, although it is necessary to consider the MDC when examining whether or not the change in gait time obtained following intervention is clinically significant, the MDC of the 10MWT in stroke patients is only reported for gait speed, and it is difficult to calculate the MDC of gait time, from the MDC of gait speed. It is also not possible to determine whether the clinical effect obtained in intervention studies is a change beyond the error range.

In a previous study of gait speed in patients with stroke, gait speed was used to classify the ability to move around in the home and outdoors (9). Classification of gait speed has also been reported to be clinically meaningful, as reports using these classifications have revealed differences in motor function and quality of life in groups classified according to these gait speeds (27). Although previous studies on the MDC of gait speed in patients with stroke have also reported that the MDC of gait speed varies depending on the baseline gait speed of the subject (28), no report has yet mentioned the MDC of gait time according to baseline gait speed in patients with stroke.

Therefore, in this study, we assessed MDCs of gait time and gait speed in the 10MWT in patients with stroke classified according to their gait speed, and examined the differences in MDCs of gait time and gait speed.

## 2. Materials and methods

### 2.1. Participants

A total of 84 stroke patients were enrolled at the Ukai Rehabilitation Hospital of the Keizankai Medical Corporation in this study (average age, 68.5 ± 13.7 years; males, *n* = 42; females, *n* = 42; average time since stroke, 75.7 ± 34.5 days; hemorrhage, *n* = 39; infarction, *n* = 45; right hemiplegia, *n* = 47; left hemiplegia, *n* = 37; Table 1).

**Table 1:** Characteristics of the total cohort and the three groups.

	Overall (*n* = 84)	Low-speed group (*n* = 19)	Moderate-speed group (*n* = 29)	High-speed group (*n* = 36)
Age (years)	68.5 ± 13.7	69.6 ± 12.0	68.0 ± 14.7	68.1 ± 14.1
Sex (male/female)	42/42	9/10	9/20	24/12
Height (cm)	161.7 ± 9.1	159.7 ± 11.2	163.6 ± 8.8	161.6 ± 7.9
Weight (kg)	59.9 ± 12.5	56.8 ± 12.7	62.5 ± 13.7	59.4 ± 11.2
Time since stroke (days)	75.7 ± 34.5	93.4 ± 40.5	72.3 ± 32.6	68.0 ± 29.9
Type of stroke (hemorrhagic/infarction)	39/45	11/8	10/19	18/18
Paretic side (right/left)	47/37	9/10	15/14	23/13
Use of ankle-foot orthosis	34	17	17	0
Use of cane (T-cane/Q-cane)	26/13	8/11	18/2	1/0
Brunnstrom recovery stage (point)	4 (2–6)	3 (2–5)	4 (3–6)	6 (5–6)
Comfortable 10-meter walking test (s)	21.14 ± 18.71	49.38 ± 21.99	16.94 ± 2.97	9.62 ± 1.48
Comfortable walking speed (m/s)	0.72 ± 0.35	0.24 ± 0.10	0.61 ± 0.09	1.07 ± 0.16
Maximum 10-meter walking test (s)	16.37 ± 13.97	40.10 ± 14.35	14.11 ± 3.56	7.58 ± 1.49
Maximum walking speed (m/s)	0.93 ± 0.48	0.28 ± 0.11	0.75 ± 0.15	1.37 ± 0.28

Data are expressed as the mean ± standard deviation or median (minimum-maximum).

Inclusion criteria were defined as the ability to walk independently without the assistance of physical therapists, with or without the use of walking aids (i.e., T-cane and Q-cane) or an ankle foot orthosis (AFO) when walking. Exclusion criteria were the presence of orthopedic diseases, significant ataxic symptoms, and aphasia or dementia leading to patient inability to comprehend the study purpose. Previous studies have reported that, based on gait speed, people can be classified as those capable of household ambulation (<0.4 m/s), limited community ambulation (0.4 to 0.8 m/s), or full community ambulation (>0.8 m/s) (9); additionally, the classification of gait speed has been reported to be clinically meaningful, as reports using these classifications have revealed differences in the quality of life in each group according to these gait speed classifications (27). Based on these previous studies, patients were divided into three groups according to gait speed: a low-speed group with a gait speed of less than 0.4 m/s; a moderate-speed group with a gait speed between 0.4 m/s and 0.8 m/s; and a high-speed group with a gait speed of 0.8 m/s or higher. All participants provided their informed consent before the start of the study. This study was conducted according to the guidelines of the Declaration of Helsinki and was approved by the Ethics Committee of Ukai Rehabilitation Hospital (approval number: 4-0040).

### 2.2. Assessments

The 10MWT is a method of assessing gait ability that has been used to evaluate a variety of conditions in patients with stroke (29). It measures speed during a 10-meter walk, although currently, there are no clear rules regarding acceleration and deceleration intervals before and after the 10-meter segment. In the present study, an acceleration distance of 3 m and a deceleration distance of 3 m was set up at the front and rear end, respectively, of the 10 m track, and gait speed was calculated by measuring the gait time in the 10 m middle section, excluding the acceleration and deceleration areas, with a digital stopwatch (30). For the assessment of parameters at a comfortable walking speed, participants were instructed to “Walk at your normal, comfortable pace”; for the fast speed, participants were instructed to “Walk as fast as you safely can.” If necessary, participants used their walking aids (i.e., T-cane and Q-cane) or AFO, although they did not receive the physical therapists’ assistance. Measurements were obtained twice at a comfortable speed and twice at the maximum speed. In addition, participants took a 20-s break between each measurement (31);.

Further, motor function was assessed using the lower-limb Brunnstrom Recovery Stage (BRS). The BRS evaluates lower extremity function on a 6-point scale, with higher scores (range: 1–6) indicating better motor function (32).

### 2.3. Data and statistical analysis

The mean of the first and second 10MWTs for each subject and the difference between the first and second measurements were used for statistical analysis as the difference between the two measurements. First, the normality of the variables obtained from the 10MWT at each speed was confirmed by the Shapiro–Wilk test. Reliability refers to the error of the measurement due to variability between study subjects, and is used to determine the discriminative ability of the measurement at a group level (33, 34). Reliability and agreement of an outcome are both essential when determining the effects of treatment in a patient population (33). In this study, to test the reliability of the first and second measurements, the inter-rater reliability of the 10MWT was assessed using a two-way mixed effect, consistency, single-rater measurement model (3, 1) intraclass coefficient (ICC) with absolute agreement, as described in previous research (31, 35). The ICCs were classified using the following categories: <0.5 = poor reliability; 0.5 < 0.75 = Moderate reliability; 0.75–0.9 = Good reliability; >0.9 = Excellent reliability (36). Then, Bland–Altman analysis was used to check for systematic bias (37). For Bland–Altman plot, see Supplementary material. In systematic bias, a fixed bias can be considered to be absent if the 95% confidence interval of the difference between two measurements includes 0 (37). Furthermore, proportional bias can be determined by testing the difference between two measurements and the correlation between two average data groups (38). When the absence of systematic bias was confirmed, the measurement error was calculated in each group using the MDC, because the difference between multiple measurements can be limited to random error. The MDC indicates the marginal range in which the change between two measurements obtained by repeated measurements, such as retests, is due to measurement error, and MDC95, the 95% confidence interval of the MDC, is generally used (39). MDC95 is calculated using the following equation:

MDC95=SEM×1.96×√2

Although several methods for calculating the standard error of measurement (SEM) included in the MDC formula have been reported (38–40), the differences between the SEM calculated by these methods and the respective MDC values calculated using these SEMs are reported to be negligible, including in clinical applications [32]. In the present study, the standard deviation of the difference between the two measurements (SDd), obtained using the following equation, was used, as reported in a previous study (41):

SEM=SDd/√2

From the above equations, the MDC of gait time and gait speed in the 10MWT, measured at the CGS and MGS in each group, were calculated.

Age, sex, height, weight, time since the stroke, type of stroke, paretic side, and BRS of the three groups were compared using a one-way analysis of variance or the Kruskal-Wallis test. The Bonferroni method was used for multiple comparisons. All statistical analyses were conducted using SPSS version 28.0 (IBM Corp., Armonk, NY, USA). Values of *p* < 0.05 were considered to indicate significance.

## 3. Results

Overall, 84 individuals with stroke were analyzed in this study (Table 1). The low-speed group comprised 19 individuals (<0.4 m/s), the moderate-speed group comprised 29 individuals (0.4–0.8 m/s), and the high-speed group comprised 36 individuals (>0.8 m/s). The results of the multiple comparison tests showed that the low-speed group had a lower BRS and severe paralysis compared to the moderate-and high-speed groups (*p* < 0.05; Table 1). No significant differences were found between the groups for the other variables. In each group, the MDC for gait time and speed were different (Tables 2, 3). The ICCs (3, 1) of the 10MWT in each group were in the ‘good’ to ‘excellent’ reliability range for both gait time and gait speed (Tables 2, 3).

**Table 2:** Minimum detectable changes in each gait speed group at comfortable speeds and reliability of repeated measurements.

Group	10-meter walking test	Outcomes	ICC (3, 1)	Bland–Altman analysis			SDd	SEM	MDC95
				Fixed bias	Proportional bias				
				95% Confidence interval	Correlation coefficient	*p* value			
Low speed group (*n* = 19)	Gait time (s)	21.14 ± 18.71	0.95	−0.56 - 2.03	−0.54	*p* > 0.05	2.68	1.89	5.25
	Gait speed (m/s)	0.72 ± 0.35	0.94	−0.01 - 0.03	1.99	*p* > 0.05	0.03	0.02	0.05
Moderate speed group (*n* = 29)	Gait time (s)	16.94 ± 2.97	0.93	−0.07 - 1.03	0.22	*p* > 0.05	1.40	1.00	2.83
	Gait speed (m/s)	0.61 ± 0.09	0.88	−0.01 - 0.04	−0.26	*p* > 0.05	0.06	0.04	0.11
High speed group (*n* = 36)	Gait time (s)	9.62 ± 1.48	0.92	−0.24 - 0.30	−0.24	*p* > 0.05	0.80	0.60	1.58
	Gait speed (m/s)	1.07 ± 0.16	0.89	−0.03 - 0.04	−0.17	*p* > 0.05	0.11	0.08	0.21

Data are expressed as the mean ± standard deviation. ICC, intraclass correlation coefficients; SDd, standard deviation difference; SEM, standard error of measurement; MDC95, Minimal detectable change.

**Table 3:** Minimum detectable changes in each gait speed group at maximum speeds and the reliability of repeated measurements.

Group	10-meter walking test	Outcomes	ICC (3, 1)	Bland–Altman analysis			SDd	SEM	MDC95
				Fixed bias	Proportional bias				
				95% Confidence interval	Correlation coefficient	*p* value			
Low speed group (*n* = 19)	Gait time (s)	16.37 ± 13.97	0.95	−2.58 - 0.10	−1.49	*p* > 0.05	3.70	2.62	7.26
	Gait speed (m/s)	0.93 ± 0.48	0.95	−0.01 - 0.01	−0.34	*p* > 0.05	0.02	0.01	0.04
Moderate speed group (*n* = 29)	Gait time (s)	14.11 ± 3.56	0.96	−0.78 - 0.19	−0.89	*p* > 0.05	1.27	0.89	2.48
	Gait speed (m/s)	0.75 ± 0.15	0.95	−0.04 - 0.01	0.77	*p* > 0.05	0.06	0.04	0.12
High speed group (*n* = 36)	Gait time (s)	7.58 ± 1.49	0.95	−0.43 - 0.01	−0.10	*p* > 0.05	0.65	0.46	1.28
	Gait speed (m/s)	1.37 ± 0.28	0.93	−0.06-0.01	0.28	*p* > 0.05	0.10	0.07	0.19

Data are expressed as the mean ± standard deviation. ICC, intraclass correlation coefficients; SDd, standard deviation difference; SEM, standard error of measurement; MDC95, minimal detectable change.

### 3.1. MDC of the 10MWT at a comfortable gait speed

MDCs in the 10MWT at a comfortable gait speed (CGS) were different in each group. The MDCs of each group at CGSs were: low-speed group: gait time 5.25 s, gait speed 0.05 m/s; moderate-speed group: gait time 2.83 s, gait speed 0.11 m/s; and high-speed group: gait time 1.58 s, gait speed 0.21 m/s (Table 2). On the 10MWT at a CGS, the slower the participant’s gait speed, the larger the error in gait time and the smaller the error in gait speed.

### 3.2. MDC of the 10MWT at a comfortable gait speed

Similar to the 10MWT at a CGS, the MDCs of the 10MWT at maximum gait speed (MGS) were also different for each group. The MDC of each group on the 10MWT at the MGS were: low-speed group, gait time 7.26 s, gait speed 0.04 m/s; moderate-speed group, gait time 2.48 s, gait speed 0.12 m/s; and high-speed group, gait time 1.28 s, gait speed 0.19 m/s (Table 3). Similar to the 10MWT at a CGS, the 10MWT at the MGS also showed that the error in gait time increased and the error in gait speed became smaller as the participant’s gait speed became slower.

## 4. Discussion

The purpose of this study was to classify both gait time and gait speed on the 10MWT in patients with stroke classified according to the participants’ gait speed, and to calculate their MDC. The results of this study showed that the MDC of gait speed measured on the 10MWT was lower in the low-speed group than in the high-speed group. The MDC results for gait speed on the 10MWT in the present study were generally similar to, and thus supported, those of the previous study (28, 42). On the other hand, the results of the present study showed that the MDC of gait time measured on the 10MWT was lower in the high-speed group than in the low-speed group. When selecting outcome measures to assess changes in gait ability in patients with stroke, clinicians and therapists need to use tools with sound psychometric properties. However, measuring outcomes in post-stroke individuals can be challenging due to symptom heterogeneity, variability in severity, and the variety of etiologies (43). The results of the present study can be used to measure changes in gait ability in patients with stroke classified according to their gait speed. Clinicians and therapists should use MDC values established from participants with similar characteristics when attempting to determine if a true change in gait speed has occurred. As areas of further research, the characteristics of patients with stroke might need to be considered when using the MDC.

On the other hand, the results of this study showed that the MDC of gait speed was different from the MDC of gait time, and slower gait speed was associated with a greater MDC. It has been shown that the lower the motor function of stroke patients, the lower their gait ability (1, 44, 45). Furthermore, previous studies have shown that the lower the gait ability, the greater the variability in gait cycle time, swing time of gait, and stride length during gait (46, 47). The present study showed lower motor function in the low-speed group compared to the high-speed group, supporting previous studies. Therefore, it was thought that the MDC of gait time in multiple measurements might have been larger in the low-speed group.

In the case of gait speed measured on the 10MWT, however, the MDC was larger in the high-speed group. Gait speed is calculated by dividing the distance covered by the gait time. Therefore, even if a large change occurs in the low-speed group, the change is small after standardization because the denominator is the long gait time. On the other hand, in the case of the high-speed group, since the denominator of gait time is short, MDC measurements are sensitive to even a slight change in gait speed, reflecting a large change when the speed is standardized. The above results suggest that the variability in calculated gait speed is larger in the high-speed group and that MDC might also be larger in the high-speed group.

Furthermore, in this study, the MDC of gait speed and gait time were calculated at both comfortable and maximum speeds. As a result, the MDC of gait speed was higher in the high speed group and the MDC of gait time was higher in the low speed group, regardless of the speed condition at the time of measurement in stroke patients. Under the 10 MWT speed condition, there was no difference in both gait speed and gait time MDC. It has been reported that the difference between comfortable gait speed and maximum gait speed is smaller in stroke patients than in normal subjects because stroke patients are unable to tolerate changes in gait speed due to their reduced balance ability caused by motor paralysis and muscle weakness (48). It is possible that these effects did not cause differences in MDC values between the 10MWT speed conditions in this verification.

The results obtained in this study, in which the MDC of gait speed and gait time in the 10MWT differed for each gait speed, suggest that the MDC depends on the gait speed of patients with stroke, and that the results should be interpreted according to their gait speed when determining changes in gait speed in response to interventions. Comparing the MDC of gait speed reported in the previous study (5, 28, 48) and the MDC of the low speed group obtained in this verification, the low speed group shows lower values than those reported in the previous study. Therefore, based on the MDC used in previous studies, any attempt to measure or capture pre-and post-intervention changes in low-velocity stroke patients would be evaluated as changes that do not exceed the MDC. Furthermore, the MDC of gait time at 10 MWT in the low speed group showed a large value, suggesting that the actual change in time may be large before and after the measurement and intervention. Conversely, the MDC of the high speed group shows larger values compared to the MDC reported in previous studies. Therefore, about high speed patients with stroke, there is a risk of judging that the MDCs reported in previous studies are changing before and after measurement or intervention, even though they are within the range of measurement error when compared before and after measurement or intervention using the MDCs reported in previous studies. Thus, the MDC of gait speed by gait speed obtained in this study may help to more accurately capture measurement and intervention changes in future studies of patients with stroke. Furthermore, while error values are sometimes used to estimate sample size (49, 50), the finding that the MDC differed depending on the subject’s gait speed in this study may help in estimating sample size in future studies.

In addition, at clinical situations, the MDC of gait time according to gait speed should also be taken into consideration, since gait time obtained with the 10MWT is often used for easy assessment of the changes in the subject’s gait ability (23–25). In recent years, intervention studies for patients with severe stroke have been reported (24, 26, 51). The results of this study suggest that, with respect to patients with low speed with severe stroke, MDC of gait time may be effective in sensitizing them to changes. Furthermore, the results presented in this study could aid future studies using gait time in the 10 MWT as an outcome.

One limitation of this study is the inter-patient variability in the time interval between stroke onset and performing the 10MWT in this study. Since the number of days elapsed since stroke onset might have at least some effect on MDC, these factors should be taken into account in the future. Another limitation is that the number of participants in each speed group varied, with the high-speed group having a larger number of participants than the low speed group. In the future, it would be desirable to calculate MDC with a larger number of participants. Furthermore, in the present validation study, the 10MWT was performed with or without the use of a walking aid, although the use of a walking aid might also affect the MDC. These limitations will need to be considered in future studies.

## Data availability statement

The raw data supporting the conclusions of this article will be made available by the authors, without undue reservation.

## Ethics statement

The studies involving human participants were reviewed and approved by the Ethics Committee of Ukai Rehabilitation Hospital (approval numbers: 4-0040). The patients/participants provided their written informed consent to participate in this study.

## Author contributions

YH and MK contributed to the study design, data acquisition, analysis, interpretation of results, and manuscript drafting. TK, KS, and MY contributed to data acquisition and interpretation, and editing of the manuscript. All authors contributed to the article and approved the submitted version.

## Funding

This research was supported by AMED under Grant Number JP22he2202017.

## Conflict of interest

The authors declare that the research was conducted in the absence of any commercial or financial relationships that could be construed as a potential conflict of interest.

## Publisher’s note

All claims expressed in this article are solely those of the authors and do not necessarily represent those of their affiliated organizations, or those of the publisher, the editors and the reviewers. Any product that may be evaluated in this article, or claim that may be made by its manufacturer, is not guaranteed or endorsed by the publisher.

## Supplementary material

The Supplementary material for this article can be found online at: https://www.frontiersin.org/articles/10.3389/fneur.2023.1219505/full#supplementary-material
