# Supplementary material for: Estimation of minimal detectable change in the 10-meter walking test for patients with stroke: a study stratified by gait speed
Source: Front Neurol. 2023 Jul 19;14:1219505. doi: 10.3389/fneur.2023.1219505 (PMC10395330; doi:10.3389/fneur.2023.1219505)
Supplement: Supplementary file 1 [file Data_Sheet_1.docx]

Supplementary Material

Estimation of minimal detectable change in the 10-meter walking test for patients with stroke: A study stratified by gait speed

**Yuichiro Hosoi ^1^, Takayuki Kamimoto ^1^, Katsuya Sakai ^2^, Masanari Yamada ^3^ and Michiyuki Kawakami ^1*^**

^1^ Department of Rehabilitation Medicine, Keio University School of Medicine, Tokyo, Japan

^2^ Department of Physical Therapy, Faculty of Health Sciences, Tokyo Metropolitan University, Tokyo, Japan

^3^ Department of Rehabilitation, Ukai Rehabilitation Hospital, Aichi, Japan

*** Correspondence:**Corresponding Author: Michiyuki Kawakami
michiyukikawakami@hotmail.com

#
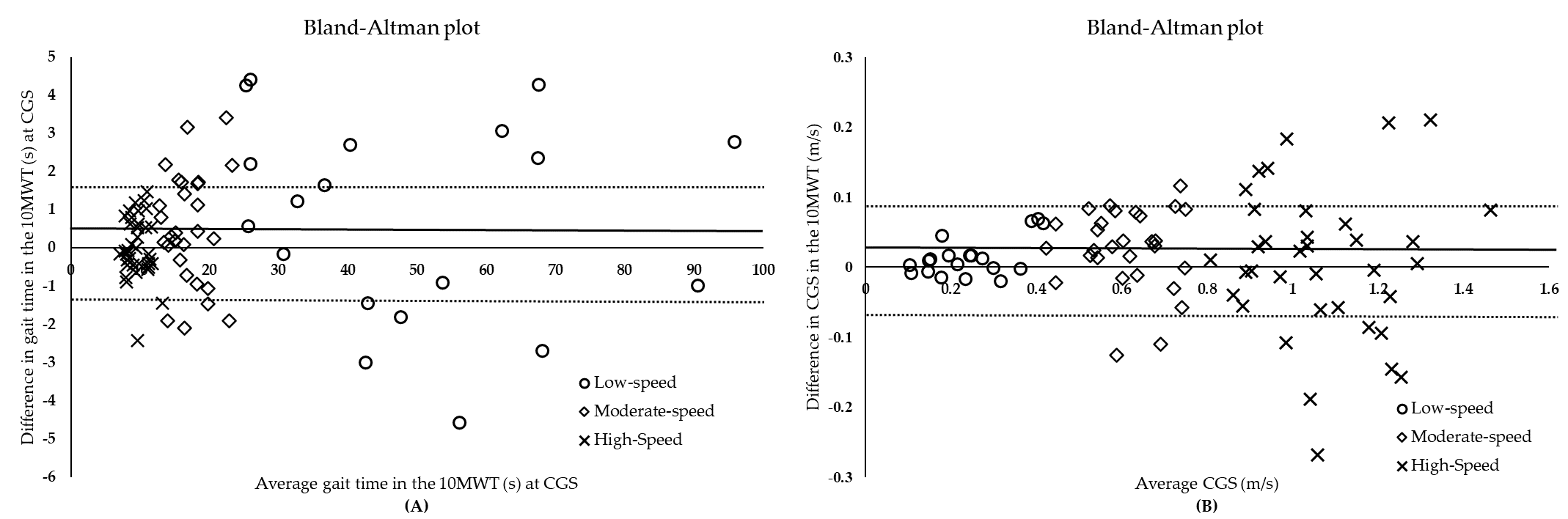
Supplementary Figures

**Supplementary Figure 1.** (A) Bland-Altman plot of gait time on the 10MWT at a comfortable gait speed (CGS). The Bland-Altman plot shows greater variability in the low-speed group compared to the high-speed group. The thick solid line represents the mean difference between sessions, and the dashed lines represent the limits of agreement. (B) Bland-Altman plot of gait speed on the 10 MWT at a comfortable gait speed. The Bland-Altman plot shows greater variability in the high-speed group compared to the low-speed group. The thick solid line represents the mean difference between sessions, and the dashed lines represent the limits of agreement.


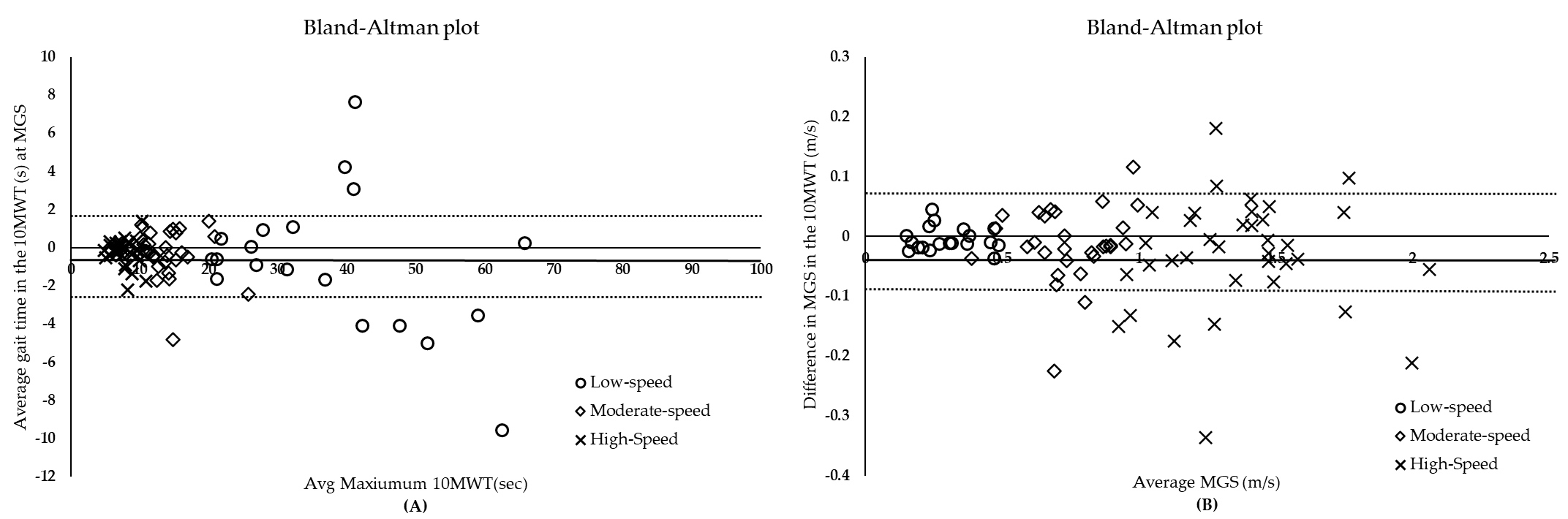


**Supplementary Figure 2.** (A) Bland-Altman plot of gait time on the 10 MWT at a maximum gait speed (MGS). The Bland-Altman plot shows greater variability in the low-speed group compared to the high-speed group. The thick solid line represents the mean difference between sessions, and the dashed lines represent the limits of agreement. (B) Bland-Altman plot of gait speed on the 10 MWT at the maximum gait speed. The Bland-Altman plot shows greater variability in the high-speed group compared to the low-speed group. The thick solid line represents the mean difference between sessions, and the dashed lines represent the limits of agreement.
